# Targeting protein–protein interactions in complexes organized by A kinase anchoring proteins

**DOI:** 10.3389/fphar.2015.00192

**Published:** 2015-09-08

**Authors:** Ana I. Calejo, Kjetil Taskén

**Affiliations:** ^1^Biotechnology Centre, University of OsloOslo, Norway; ^2^Centre for Molecular Medicine Norway, Nordic European Molecular Biology Laboratory Partnership, University of Oslo and Oslo University HospitalOslo, Norway

**Keywords:** cAMP, AKAP, protein–protein interaction, heart, disruptor peptide, small molecule

## Abstract

Cyclic AMP is a ubiquitous intracellular second messenger involved in the regulation of a wide variety of cellular processes, a majority of which act through the cAMP – protein kinase A (PKA) signaling pathway and involve PKA phosphorylation of specific substrates. PKA phosphorylation events are typically spatially restricted and temporally well controlled. A-kinase anchoring proteins (AKAPs) directly bind PKA and recruit it to specific subcellular loci targeting the kinase activity toward particular substrates, and thereby provide discrete spatiotemporal control of downstream phosphorylation events. AKAPs also scaffold other signaling molecules into multi-protein complexes that function as crossroads between different signaling pathways. Targeting AKAP coordinated protein complexes with high-affinity peptidomimetics or small molecules to tease apart distinct protein–protein interactions (PPIs) therefore offers important means to disrupt binding of specific components of the complex to better understand the molecular mechanisms involved in the function of individual signalosomes and their pathophysiological role. Furthermore, development of novel classes of small molecules involved in displacement of AKAP-bound signal molecules is now emerging. Here, we will focus on mechanisms for targeting PPI, disruptors that modulate downstream cAMP signaling and their role, especially in the heart.

## Introduction

**FIGURE 1:**
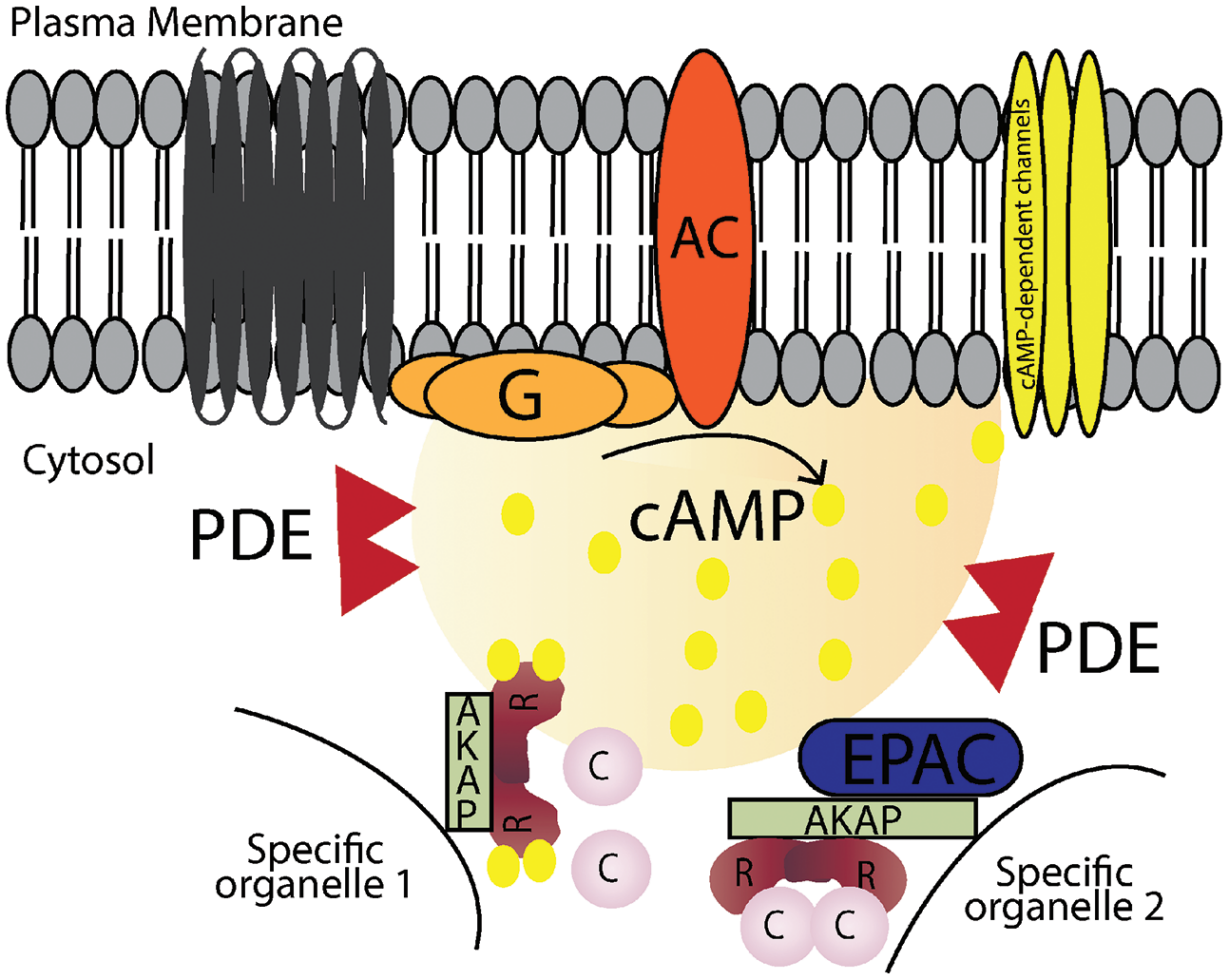
**Schematic illustration of cAMP signaling pathways.** Stimulation of G-protein-coupled receptors leads to activation of adenylyl cyclase (AC), which converts ATP into cAMP. cAMP increases in local microdomains and binds to different effectors such as protein kinase A (PKA), cyclic nucleotide gated ion channels and exchange protein directly activated by cAMP (Epac) leading to specific downstream effects. Cyclic nucleotide phosphodiesterases (PDEs) hydrolyse cAMP into AMP and terminates the signal. A-kinase anchoring proteins (AKAPs) anchor the signaling molecules involved in the pathway and target them to specific organelles in the cell.

Intracellular 3′-5′-cyclic adenosine monophosphate (cAMP) is an important second messenger that regulates a number of biological processes. Even though cAMP is diffusible, its concentration and signaling are tightly controlled and coordinated through the involvement of a molecular machinery coordinating the spatial and temporal processes of localized cAMP signaling events. Signal transduction through the cAMP pathway starts by stimulation of G-protein-coupled-receptors (GPCRs), via specific extracellular ligands leading to activation of adenylyl cyclase (AC), which converts ATP into cAMP. The rise in intracellular cAMP levels leads to a set of events mediated by specific effector molecules, hereunder protein kinase A (PKA; Walsh et al., 1968), cyclic nucleotide gated ion channels (Brown et al., 1979) and exchange protein directly activated by cAMP (Epac; de Rooij et al., 1998; Kawasaki et al., 1998). To terminate the signal, intracellular cAMP levels must be brought back to basal levels; this is attained by cyclic nucleotide phosphodiesterases (PDEs), which hydrolyse cAMP and/or cGMP. Additionally, cAMP signalosomes targeted to specific subcellular locales by A-kinase anchoring proteins (AKAPs) bring together signal initiators, effector and terminators in supramolecular signaling complexes. The existence of these specific complexes (illustrated in **Figure 1**) governed by protein–protein interactions (PPIs) creates an opportunity for new therapeutic strategies to control cAMP dependent signaling that is out of tune or involved in pathologies. In this review we will first focus on signaling through AKAP-coordinated complexes, next on targeting PPIs as a possible strategy to control and regulate cAMP signaling events and finally mention a few examples of possible PPIs that could be targeted. We will discuss cAMP/PKA/AKAP signaling in general terms but will particularly focus on the heart, where cAMP signaling pathways are involved in different stages of the cardiac cycle and in several pathologies.

## cAMP Compartmentation

Cyclic AMP is a ubiquitous intracellular second messenger involved in the regulation of a wide variety of cellular processes. For this reason there are molecular mechanisms that rigorously control cAMP signal responses, intimately linked with spatial and temporal fine tuning. The first indication that cAMP could be organized in specific microdomains came in the early 1980s, when different GPCR ligands were observed to trigger very different cardiomyocytes responses, even if there was a similar increase in cAMP levels (Hayes et al., 1980). Meanwhile, the development of new methodologies to study compartmentation (for example, fluorescent resonance energy transfer FRET, reviewed in Zaccolo and Pozzan, 2002; Smith et al., 2006) provided solid evidence of cAMP dynamics and of the mechanisms and proteins involved.

First, an important starting point for generation of well-defined local gradients of cAMP is the organization and diverse expression of receptors, G proteins and associated cyclases at the plasma membrane. The G proteins are heterotrimeric, guanosine triphosphate-binding (GTP-binding) proteins, assembled from three subunits: α-, β- and γ-. There are more than 20 different Gα-subunits that can activate or inhibit different effectors. For example, Gαs activates and Gαi inhibits ACs, respectively. Like G proteins, AC exist in different isoforms in mammals, most of them are associated with the plasma membrane (AC1-9) whereas AC10 is soluble (Steegborn, 2014). Both G proteins and AC isoforms have also been reported in lipid rafts and caveolae, and implicated in the generation of local cAMP microdomains at the membrane (Ostrom and Insel, 2004; Patel et al., 2008). Additionally, it was recently shown that cAMP production does not exclusively occur at the plasma membrane and is not terminated when receptors are internalized (Calebiro et al., 2009; Ferrandon et al., 2009). Moreover, Irannejad et al. (2013) have shown GPCR signaling both in the plasma membrane and after internalization in living cells using a biosensor. In summary, both G proteins and ACs have a distribution that contributes to generation of local gradients of cAMP.

Second, the expression and availability of various effector molecules in cAMP microdomains also contribute to discretely controlling how the signal is propagated (Mei et al., 2002; Bresnnesvik et al., 2005; Bacallao and Monje, 2013; Vitali et al., 2014). The specificity introduced by different cAMP effector molecules is illustrated, for example in the heart where it was shown that Epac modulates cardiac sarcomeric contraction despite a decrease in Ca^2+^ levels, while PKA modulates contractility via an increase in intracellular Ca^2+^ (Cazorla et al., 2009). Here, we will not go into details with respect to cAMP-gated ion channels and Epac, however, cAMP effectors can perform both synergistically and antagonistically in the regulation of specific cellular functions and coordinated action may have biological significance (reviewed in Craven and Zagotta, 2006; Cheng et al., 2008). The PKA holoenzyme is a tetramer composed of regulatory (R) subunit dimer and two catalytic (C) subunits, which are associated in the inactive state when cAMP intracellular levels are low. When cAMP levels increase PKA becomes activated; this proceeds by a concerted reaction where cAMP molecules bind cooperatively to the two cyclic nucleotide binding domains (CNBD) in each R-subunit of PKA leading to a conformational change by releasing the C-subunit. The free C-subunit becomes active and can then phosphorylate specific serine/threonine residues in target proteins, usually in the sequence Arg-Arg-X-Ser/Thr, where X is a hydrophobic amino acid. There are two types of PKA holoenzymes, type I and type II, that mainly differ in their localization and affinity for cAMP and with different R-subunit composition (RI and RII) (Reimann et al., 1971; Corbin et al., 1975; Cadd et al., 1990; Dostmann and Taylor, 1991; Gamm et al., 1996). Both R-subunits are very similar concerning their domain organization which includes the N-terminal docking and dimerization (D/D) domain important for localization inside the cell, a substrate/auto-inhibitor region that binds to the C-subunit in the holoenzyme and in the C-terminal two highly conserved CNBDs (Corbin et al., 1978; Doskeland, 1978). Although, both RI and RII share the same organization their substrate/auto-inhibitor region is significantly different, in that RII can be autophosphorylated whereas RI contains a pseudo-phosphorylation site. Another difference between PKA-RI and PKA-RII is their localization, where PKA type I is primarily found in the cytosol while PKA type II is predominantly localized to specific cellular organelles (Tasken and Aandahl, 2004; Wong and Scott, 2004). Additionally, PKA type I and type II also differ from each other in their cAMP activation constants which is lower for type I than for type II (typically 50–100 nM versus 200–400 nM; Dostmann and Taylor, 1991). PKA exist in different isoform combinations encoded by different genes, four C-subunit isoforms (in human Cα, Cβ, Cγ, and PRKX) and four R-subunit isoforms (RIα, RIβ, RIIα, and RIIβ). In combination these different R- and C-subunits isoforms can form different PKA holoenzymes that can be present in diverse signalosomes with distinct expression patterns in different tissues and cell types.

Third, intracellular gradients of cAMP and consequently their signaling pathways are highly controlled by PDEs. They are key players in controlling intracellular cAMP levels due to the fact that PDEs are the only cAMP degrading enzymes. PDEs catalyze the degradation of cAMP by breaking the phosphodiesterase bound resulting in adenosine-5-monophosphate (AMP). PDEs are highly conserved between species, with around 50 different isoforms that are part of 11 families (PDE1-11) with different enzymatic and regulatory characteristics (Houslay and Milligan, 1997; Conti and Jin, 1999). They all share similarities in their structure, mainly in the C-terminal catalytic domain, while the N-terminal regulatory and targeting domains differ (Bender and Beavo, 2006; Conti and Beavo, 2007). PDEs can be cAMP-specific, cGMP-specific or hydrolyse both. The great variety in isoforms, their specific tissue and subcellular localization and the fact that PDEs have different enzymatic profiles, makes PDEs key players in spatial and temporal control of intracellular cAMP levels. In the heart, the cAMP-specific PDE4 and cAMP/cGMP PDE3 are responsible for the majority of cAMP hydrolysed in cardiomyocytes (Mongillo et al., 2004; Rochais et al., 2004; Fischmeister et al., 2006; Mika et al., 2012).

Fourth, scaffold proteins coordinate the physical assembly of components of a signaling pathway. In cAMP signaling pathways AKAPs are responsible for the assembly of specific signalosomes. They form complexes between PKAs and their specific targets in localized subcellular domains, consequently they are important contributors to compartmentation, which will be further discussed in the next chapter.

## AKAPs – Scaffold Proteins Involved Assembly of Supramolecular Signaling Complexes

**FIGURE 2:**
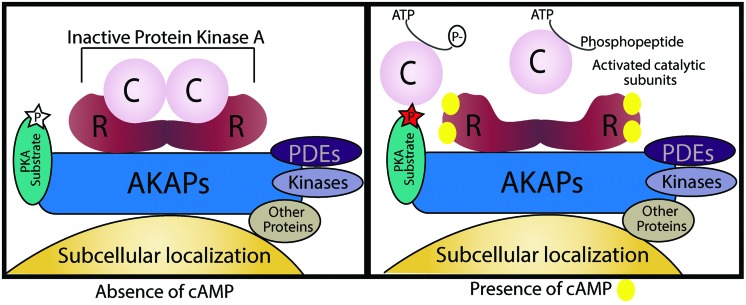
**Schematic illustration of an A kinase anchoring protein (AKAP).** AKAPs are categorized by four different characteristics. First, an amphipathic α-helical region of the AKAP interacts with D/D-domain of the PKA R-subunit dimer. Second, they target the supramolecular complex to specific subcellular localizations. Third, AKAPs may also hold PKA substrates by direct binding or by targeting in their vicinity. Lastly, AKAPs can also function as signaling scaffolds for other signaling enzymes. In the absence of cAMP, PKA is inactive and its substrates are not phosphorylated, when cAMP levels increase it binds to the R-subunits and the active catalytic subunits are free to phosphorylate their targets.

The spatial and temporal organization of cAMP/PKA signaling is attained by a carefully tuned balance between local activation of the signal effector and signal termination machinery assembled and targeted by AKAPs. There are more than 50 AKAPs identified, and even though they belong to a structurally diverse family they all share the ability to enable tightly regulated phosphorylation of substrates that are anchored to or localized in the vicinity of AKAPs together with PKA (Tasken and Aandahl, 2004; Wong and Scott, 2004). The four main features that characterize the AKAP complexes formed (**Figure 2**) are:

(1) Anchoring of the dimer R-subunit D/D-domain through interaction between the hydrophobic pocket of PKA and the 14–18 amino acid amphipathic helix region of AKAPs (Carr et al., 1991). AKAPs can bind both RI- and RII-subunits of PKA, the majority binds to RII-subunits;
(2) Targeting to specific subcellular locations. AKAPs typically contain a targeting domain that localizes the entire AKAP/PKA complex inside the cell. This can be a defined organelle, membrane or structure and the binding may occur by protein–protein or protein–lipid interactions.
(3) Directly binding to or co-localizing with specific substrates that will be phosphorylated by PKA.
(4) Assembly of multi-protein complexes with additional signaling enzymes such as PDEs, phosphatases (PPs), and other kinases;

AKAPs can be found in a number of tissues and are involved in the composition of a wide variety of complexes implicated in different signaling cascades (reviewed in Pidoux and Taskén, 2010; Tröger et al., 2012; Esseltine and Scott, 2013; Scott et al., 2013). Most of these AKAP complexes preferentially anchor the PKA type II holoenzyme with higher affinity. There are, however, some exceptions where AKAPs anchor PKA-RI. For example, the cardiomyocyte sphingosine kinase interacting protein (SKIP) exclusively binds PKA type I and facilitates phosphorylation of ChChd3 inside mitochondria (Means et al., 2011). Recently a small membrane AKAP (smAKAP) was found to target RI to the plasma membrane (Burgers et al., 2012). There are also some cases where AKAPs can have dual specificity, for example D-AKAP1 and D-AKAP2 (Huang et al., 1997a,b), ezrin (Ruppelt et al., 2007) and Opa1 (Pidoux et al., 2011) can bind both RI and RII at physiological concentrations. There is also a number of cellular contexts where AKAPs contribute to the spatial organization of other effectors such as Epac (Dodge-Kafka et al., 2005). AKAPs complexes have been shown to directly interact with PPs and PDEs which provides tight control of signal termination, since PPs that remove phosphorylation or PDEs that degrade cAMP are found in the same complex (Coghlan et al., 1995; Schillace and Scott, 1999; Dodge et al., 2001; Tasken et al., 2001).

In the heart the existence of a supramolecular complex with PKA/mAKAP/PDE4D3 creates a negative feedback loop mechanism under stimulation, where PDE4D3 phosphorylation increases cAMP hydrolysis and turns off PKA activity (Dodge-Kafka et al., 2005). Several AKAPs occur in different isoforms and spliced variants that are targeted to different subcellular localizations. For example, AKAP18 has several isoforms (α, β, γ, and δ) that are localized to distinct subcellular structures in specific cell types. Both AKAP18α and AKAP18β are mainly found in plasma membrane, while AKAP18α is associated with L-type Ca^2+^ channels in the skeletal muscle and pancreatic cells less is known about AKAP18β function (Fraser et al., 1998; Gray et al., 1998). When overexpressed in polarized epithelial kidney cells, however, AKAP18α and AKAP18β preferentially localize to the basolateral and apical membrane, respectively (Trotter et al., 1999). Overexpressed AKAP18γ cloned from pancreas and lung is mainly localized in the cytosol but it was also found in the nucleus of mouse oocytes (Trotter et al., 1999; Brown et al., 2003). AKAP18δ was first found in the kidneys anchored to vesicles (Henn et al., 2004) and later AKAP18γ/δ was found in cardiomyocytes anchored to the sarcoplasmic reticulum (SR; Lygren et al., 2007). Furthermore, AKAPs can target complexes to other localizations like mitochondria, the Golgi complex, centrosomes, cytoskeleton and many other loci (Wong and Scott, 2004; Tröger et al., 2012).

Several AKAPs have been found to be expressed in cardiac tissue involved in different processes: calcium-induced calcium release in depolarization and plateau phase, cardiac repolarization and cardiac remodeling due to stress responses (Scott et al., 2013; Soni et al., 2014). It has been shown that different pathologies are associated with AKAPs, due to polymorphisms and mutations in members of this family of proteins, in heart diseases (Chen et al., 2007) and cancer development (Frank et al., 2008). In the heart, AKAPs have been implicated in several cardiac diseases such as rhythm disorder, long-QT syndrome, cardiac hypertrophy and heart failure (reviewed in Soni et al., 2014).

This review will focus on the potential targeting of AKAPs as a therapeutic strategy.

## Targeting Protein–Protein Interactions

There are a number of approaches to target signal effector and signal termination enzymes in specific signalosomes. One strategy is the development of inhibitors against specific enzymes and receptors. However, as signaling enzymes may be components of several different signalosomes, specificity may not be at the level of the individual type of complex. Another possibility would be to target scaffold protein such as AKAPs using RNA interference (RNAi). Small interfering RNAs (siRNAs) can be specifically designed to target any gene and can silence target mRNA expression to overcome different pathologies. Even though siRNA is been increasingly used and some siRNA based therapies are in human clinical trials, siRNA therapeutics still need to overcome the obstacles of efficient drug delivery to be a fully viable drug development strategy (for more details about this approach see de Fougerolles et al., 2007). In this review we will mainly focus on another approach, targeting AKAPs by disrupting the binding between two proteins, i.e., interfering with PPIs.

Hundreds of thousands of PPIs probably occur in human cells and are involved in assembly of supramolecular signaling complexes and signalosomes as well as in target-depend signaling by docking and adaptors. PPIs represent an exciting group of potential therapeutic targets implicated in a wide array of diseases. The fact that they are intrinsically associated with specific signalosomes offers potential for high specificity and PPIs may therefore constitute valuable targets in new therapeutic strategies. For this reason designing and developing PPI disruptor peptides represents an area of increasing interest in target validation and drug discovery. Peptides can be highly selective and specific and their affinity to the target makes them appealing candidates in vivo due to minimal off-target effects. Peptides and peptidomimetics offer several benefits such as ease of synthesis, optimization and evaluation, high affinity, minimal immune responses and low toxicity. However, peptides can be metabolically cleaved and rapidly cleared from body and non-natural peptides or peptidomimetics that abandon the amino acid backbone may be necessary to avoid excessive degradation. Furthermore, intracellular delivery may be an issue (reviewed in Cochran, 2000). Nevertheless, the development of disruptor peptides for PPIs can be valuable research tools to perturb supramolecular signaling complexes and retrieving information regarding the role of the proteins involved.

During this time also disruptor peptides for complexes involving AKAPs have been progressing, mainly to prevent the interaction between AKAP and PKA. These peptides were primarily designed to serve as tools to study AKAPs and interaction with different PKA R-subunits, both *in vitro* and *in situ* inside cells. As mentioned above, AKAPs bind through a conserved amphipathic helixes domain to the hydrophobic dimerization domain of the PKA R-subunit (Carr et al., 1991). These small disruptor peptides mainly mimic the amphipathic helices domain of AKAP, like in the case of Ht31, the first and most commonly used disruptor peptide. This was derived from the PKA binding domain in AKAP-Lbc (Carr et al., 1992) and it was later shown to be non-selective, with the ability to disrupt both RI and RII from AKAPs (Herberg et al., 2000). More than 20 years later Ht31 is still being used as a tool to describe new AKAPs, for example neurochondrin (Hermann et al., 2015). In 2003, a disruptor peptide was developed by determining the minimal binding domains of several AKAPs that had high binding affinity to RII, called AKAP-in silico (AKAP-IS; Alto et al., 2003). A later version was developed to have almost no RI-binding, SuperAKAP-IS (Gold et al., 2006). At the same time, peptides designed to disrupt AKAP/RI binding, like PV38 (Burns-Hamuro et al., 2003) and RIAD (Carlson et al., 2006) were put in use. Besides the improvements regarding binding affinity to distinguish between AKAP binding to RI or/and RII, a big limitation is the cell permeability of the designed peptides. Several approaches have been used to address this problem by attaching a cell-permeable poly-basic sequence to the disruptor peptide, like the TAT or *antennapedia* sequences or a poly-arginine peptide. For example, a peptide derived from the TAT protein of the human immunodeficiency virus (HIV-1) can, when linked to the disruptor peptide, easily facilitate its transport into the cell. TAT-AKAP-IS could at micromolar concentrations disrupt endogenous AKAP/PKA interaction and affect PKA subcellular localization in insulin-secreting pancreatic B-cells (Faruque et al., 2009). In perfused hearts, TAT-conjugated A-kinase-anchoring disruptor (TAT-AKAD) affected heart rate and contractility after β-adrenergic stimulation and disrupt PKA localization in cardiomyocytes (Patel et al., 2010). Another approach that has been developed to improve peptide permeability is to use all-hydrocarbon-stapled α-helical peptides, where non-natural amino acids are incorporated into the peptide resulting in a stapled peptide that is locked in a α-helical conformation. Additionally, it has been shown that stapled peptides have increased binding affinity, less susceptibility to proteolytic degradation, improved pharmacologic performance and serum half-life (Verdine and Hilinski, 2012). Using this technique, Wang and co-workers developed disruptor peptides that are highly cell permeable to different cell-lines and that could efficiently prevent the interaction between AKAP and PKA, which they called Stapled Anchoring Disruptors (STADS). They designed STAD-2 and STAD-3 (Wang et al., 2014) and RI-STAD-1 and RI-STAD-2 (Wang et al., 2015) that are highly selective for disrupting the interaction between AKAPs/PKA-RII and AKAP/PKA-RI, respectively. Interestingly, STAD-2 has been used in a very different cell model system as a potential strategy to study and develop new antimalarials targets (Flaherty et al., 2015).

Even if these are highly efficient peptides, the fact that they disrupt the AKAP/PKA interaction diminishes their specificity since this interaction is common to all PKA-AKAP complexes. By disrupting the AKAP/PKA interaction, these peptides will affect several AKAP/PKA complexes inside the cell regardless of the AKAP present. A much more precise strategy would be to disrupt individual AKAP complexes, which would be possible by preventing the interaction between a specific AKAP and an attached substrate protein that will be phosphorylated by PKA. There are several examples of such peptides, which were also designed mainly as tools to study and confirm AKAP-interactions. One example is a short peptide derived from the phospholamban (PLB) domain that binds to AKAP18δ and competes with and displaces the AKAP18δ/PLB interaction (Lygren et al., 2007). This PLB peptide with a poly-arginine attached to the C- or N-terminus for penetrance in neonatal cardiomyocytes, blocked noradrenalin-induced increase in Ca^2+^ reabsorption. Another possibility would be to disrupt the interaction between the AKAP targeting domain and its interaction partner providing subcellular localization. For example Dodge-Kafka et al. (2005) designed a fragment encoding residues 585–1286 of mAKAP that displaced mAKAP from the perinuclear membrane.

Recently, a new approach was established that uses structure-based phage selection to design new RII D/D domain fragments that can selectively distinguish between different AKAPs, which were designated R_Select_ (Gold et al., 2013). Using Western blot and amplified luminescence proximity homogenous assay (AlphaScreen) assays R_Select_AKAP2 and R_Select_AKAP18 were shown to preferably interact with AKAP2 and AKAP18 *in vitro*. Additionally, *in vivo* experiments showed that the same mutants have a similar subcellular distribution as their AKAP partners and they recognize and interact with them.

Even though disruptor peptides are of great importance for interfering with PPIs and an invaluable asset to understand cAMP/AKAP/PKA signaling effectors and effects, they have limited use as therapeutics. Peptides are designed and derived from one of the binding proteins involved. When compared to peptidomimetics and small molecules, low permeability and poor per-oral bioavailability are big drawbacks in peptides. Peptidomimetics has been used also as a possible strategy for the modulation and regulation of AKAPs by interfering with PPIs. For example, RIAD peptidomimetics have been developed by adding unnatural amino acids at different positions, leading to increased stability in serum though still retaining specificity to disruption of the AKAP/PKA-RI interaction (Torheim et al., 2009). In addition, a RIAD peptidomimetic (RIAD-P3) has been shown to limit HIV-1 viral replication and stabilize CD4 levels by disrupting AKAP/PKA-RI *in vivo* (Singh et al., 2014).

When compared to peptides, small molecules offer several advantages in drug discovery: due to their smaller size they can be synthesized easily and at a lower price, get faster to their targets, have potential for higher oral bioavailability, can offer better stability and can be used to allosterically target quite large protein interaction surfaces. However, one downside to this approach is the fact that it requires detailed and profound knowledge about the interaction between the two proteins involved and PPI targeting can be quite challenging and make small molecule approaches less attractive from a drug discovery point-of-view.

Several techniques have been contributing to identifying PPIs and increasing the information regarding their structure such as X-ray crystallography, nuclear magnetic resonance (NMR), thermal shift assay, surface plasmon resonance (SPR), immunoprecipitation and other biochemical assays as well as in silico modeling of interactions. However, there are still challenges when screening for small molecule PPI disruptors: the PPI interfaces may be big, discontinuous or flat and hydrophobic with an absence of pockets and typically a screen yields PPI disruptors with micromolar affinity. Peptides do not have these limitations, but have issues with stability and permeability. In order for small molecule targeting of PPIs to be successful, a topology of the interacting proteins with small pockets or the identification of key residues that contribute to the binding energy, so called “hot spots” are necessary requirements (reviewed in Turnbull et al., 2014). Furthermore, increasing the size and molecular weight of the small molecule or assembling new compounds by a fragment-based approach may overcome some of these problems. Small molecules can also allosterically bind to one of the two interacting proteins outside the binding interface inducing conformational changes affecting the PPI (Arkin and Wells, 2004; Fry, 2006; Wells and McClendon, 2007; Turnbull et al., 2014).

The first study reporting the use of small molecules as disruptors of PPIs in AKAP complexes was in Christian et al. (2011), when it was reported that a group of structurally similar small molecules could prevent the interaction between an AKAP and PKA-RII. After screening a library of 20,000 compounds, the authors found that FMP-API-1 and its derivatives disrupted the interaction between AKAP18δ and both RIIα and RIIβ by allosteric binding to RII outside its D/D domain instead of binding to the AKAP interacting surface. Using cardiomyocytes they showed both *in vitro* and *in vivo* evidence that these small molecules disrupt AKAP and RII binding and at the same time activate PKA, indicating a dual effect.

Increasing interest in PPI disruptors together with improvements in high-throughput screening (HTS) for compounds targeting PPIs has resulted in an increasing number of projects in this area. Combinations of primary and secondary assays in HTS can create an attractive and very useful setting to screen fast and easily large compounds libraries. These are normally miniaturized assays performed in a robotics workstation, where different instruments and liquid handling systems are used. There are several published assays to screen both for peptides and small molecules that were specifically developed to target PPIs in complexes involving AKAPs such as AlphaScreen; SPR; enzyme-linked immunosorbent assay (ELISA); and homogenous time-resolved fluorescence (HTRF) (Stokka et al., 2006; Jarnaess et al., 2008; Christian et al., 2011; Gold et al., 2013; Schächterle et al., 2015).

Certainly targeting PPIs is a very appealing and promising therapeutic strategy as it is possible to specifically target interactions in single molecules complexes at defined subcellular places that until now has not been fully address and exploited.

## Therapeutic Targets in the Heart in cAMP Signaling Pathways

Heart pathologies are a leading cause of hospitalization and mortality in the Western World. Moreover, population growth and increase in life expectancy accelerates the number of heart incidents. Currently there are several treatment choices that interfere with cAMP signaling in the heart. Beta-blockers block adrenergic signaling and have a negative inotropic and chronotropic effect. In contrast dopamine has a positive inotropic effect and paces the heart. Beta-blockers are, however, accompanied by significant side effects, because they affect all downstream signaling. As an alternative to beta-blockers, the recently described biased agonists and antagonists can provide functional selectivity, where the biased ligand activates or terminates a specific intracellular signaling pathway downstream of the GPCR. In this case one can achieve a more specific and selective effect rather than the “all or nothing” effect of beta-blockers (Kenakin and Miller, 2010). Particular attention has been given to the importance of ligand bias as a new potential therapeutic strategy for classical GPCR in cardiology targeting angiotensin II type 1 receptors and the β-adrenergic receptors (DeWire and Violin, 2011). With this strategy it might be possible to attain a more precise and specific outcome without the unwanted side effects, making these ligands very interesting drugs for new therapeutic strategies. Additionally, a more effective strategy could be to directly alter a specific AKAP signalosome by affecting a single PPI in complexes where AKAPs have well-established roles in the heart regarding: (i) Ca^2+^-handling and excitation-contraction coupling; (ii) hypertrophic stress responses; and (iii) controlling electrical signaling. Here we will shortly mention a few of the most relevant AKAPs targets.

### (i) Ca^2+^-Handling and Excitation-Contraction Coupling

In cardiac myocytes AKAP18α (also known as AKAP15) anchors PKA to L-type Ca^2+^ channels in plasma membrane. It was also shown that in skeletal muscle cells AKAP18α directly interacts with both PKA and the channel (Hulme et al., 2002). The authors used a peptide to disrupt the leucine zipper motif interaction between the AKAP and the channel, inhibiting voltage-dependent potentiation of L-type Ca^2+^ channel. The disruptor peptides abolish sympathetically induced, AKAP18α-dependent PKA phosphorylation of L-type Ca^2+^ channels and consequently channel open probability. This in turn prevents Ca^2+^ entry increased in response to local cAMP that increases contractility, resembling β-blockers effect.

More recently, has also been shown that in cardiac myocytes AKAP5 (also known as AKAP79/150) assembles a complex in caveolin 3-associated L-type Ca^2+^ channels together with β-adrenergic receptor, PKA, AC5/6 and calcineurin (CaN), which is important for sympathetic regulation (Nichols et al., 2010). Also, AKAP5 was shown to directly interact with L-type Ca^2+^ channels in HEK293 cells via modified leucine zipper motifs, similar to AKAP18α (Oliveria et al., 2007). Taken together this suggests that both AKAP18α and AKAP5 supra molecular complexes are involved in sympathetically stimulated Ca^2+^ entry trough L-type Ca^2+^ channels, likely associated with different channels subpopulation and cAMP microdomains. Additionally, Makarewich et al. (2012) showed that these caveolin 3-associated L-type Ca^2+^ channels might be an important target for cardiac hypertrophy.

**FIGURE 3:**
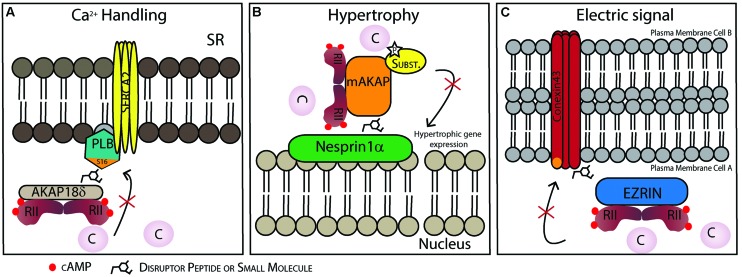
**Possible therapeutic strategies to target protein–protein interactions (PPIs) in specific AKAP complexes in the heart. (A)** Disruption of the AKAP18γ/δ-PLB interaction prevents PLB phosphorylation on Ser16 and dislocation from SERCA2. This inhibits SERCA2 activation and consequently Ca^2+^ uptake into the sarcoplasmic reticulum (SR). **(B)** Disruption of the nesprin-1α/mAKAP interaction promotes AKAP/PKA complex dissociation from the perinuclear membrane and might be a strategy to reduce hypertrophy. **(C)** Disruption of the connexin 43-ezrin interaction could prevent PKA-mediated phosphorylation increasing inter-cardiomyocyte conductivity which could be cardioprotective following myocardial infarction damage.

Lygren et al. (2007) found a PKA/AKAP18δ/PLB complex that regulates SR Ca^2+^-ATPase 2 (SERCA2) in the heart, which was also later shown in human myocardium (Ahmad et al., 2015). The PLB/SERCA2 complex plays a crucial role in calcium homeostasis in cardiomyocytes and is major regulator of cardiac contractility *in vivo* (Koss and Kranias, 1996). Under normal conditions dephosphorylated PLB inhibits SERCA2 mediated Ca^2+^-reabsorption into the SR, a process that is critical for relaxation of the cardiomyocytes and refilling of the heart before the next contraction. However, AKAP18δ acts as a scaffold protein forming a complex of AKAP18δ and PKA together with PLB/SERCA. Upon β-adrenergic stimulation, PLB is phosphorylated and inhibition of SERCA2 is released leading to an increase in Ca^2+^-reuptake into the SR allowing for pacing the heart by facilitating faster relaxation and filling. Inhibition of PLB phosphorylation by targeting this complex with PPI disruptors is thought to be cardioprotective (Lygren and Taskén, 2008). Moreover, the AKAP18δ/PLB complex should be relatively heart specific, thus minimizing potential side effects. The binding between AKAP18δ and PKA has already been targeted, both by peptides and small molecules. Here they used HTS to screen libraries of small molecules which inhibit the binding between AKAP-PKA (Christian et al., 2011). Another possibility would be to disrupt the interaction between other proteins in the complex (**Figure 3A**), Lygren et al. (2007) also showed that in neonatal cardiac myocytes the displacement of AKAP18δ/PLB by a short peptide (13–20 aa) affects the phosphorylation of PLB on Ser16 and consequently Ca^2+^- re-uptake into the SR. Also, removal/reduction of AKAP18δ by siRNA injected in adult cardiomyocytes had the same effect.

In the two previous examples the PPI disruptor approach has been to target the binding between AKAPs and the phosphorylated substrate. Another possibility is to target PPIs between AKAPs and other enzymes, such as PDEs which can potentially be good targets for therapeutic strategies since PDEs are involved in many physiological aspects of cardiomyocyte function, for review see (Mongillo and Zaccolo, 2006). PDEs contribute to the regulation of small cAMP pools by anchoring to AKAPs (Richter et al., 2008; Scott and Santana, 2010). In rat primary cardiomyocytes both PDE3 and PDE4 are likely the major contributors in cAMP signaling (Mongillo et al., 2004; Rochais et al., 2004; Fischmeister et al., 2006; Mika et al., 2012). Additionally, PDE4D are components of the supramolecular complex formed with AKAP18δ/SERCA2 (Lygren et al., 2007; Ahmad et al., 2015). Also in renal principal cells PDE4D is anchored in the AKAP18δ complex (Stefan et al., 2007). Recently, Ahmad et al. (2015) also showed that PDE3A1 also associates with the PLB/AKAP18δ/SERCA2 supramolecular signaling complex in human myocardium SR.

### (ii) Hypertrophic Stress Responses

A-kinase anchoring proteins are also present in signalosomes that govern the cellular response known as myocardial hypertrophy, which occurs due to different types of cardiac stress where increased levels of catecholamines induce transcriptional activation evoking heart remodeling. These “hypertrophy signalosomes” include a multicity of proteins: protein kinases, PDEs, PPs, calcium channels and others, that all come together in supramolecular complexes coordinated by AKAPs (reviewed in Negro et al., 2008; Carnegie and Burmeister, 2011; Diviani et al., 2013; Soni et al., 2014). At least two AKAPs are involved in cardiomyocyte hypertrophy, an alternatively spliced isoform of muscle AKAPβ (mAKAP, previously called AKAP100; Dodge-Kafka et al., 2005) and AKAP-Lbc (Appert-Collin et al., 2007).

One of the signalosomes that is scaffolded by mAKAP includes PKA, ryanodine receptors (RyR), CaN and transcription factors NFAT and MEF2 (Pare et al., 2005a; Li et al., 2010, 2013). In response to a cAMP increase due to β-adrenergic stimulation, activated PKA phosphorylates RyR channels, the Ca^2+^ release activates CaN, which mediates NFAT and MEF2 transcriptional activity. NFAT and MEF2 transcriptional activity regulated by CaN is dependent and requires mAKAP (Li et al., 2010, 2013). Additionally, Li et al. (2013) showed that cardiomyocytes expressing a mCherry-CaN binding site peptide disrupts mAKAP/CaN complex and inhibits adrenergically induced myocyte hypertrophy, providing a possible therapeutic strategy by targeting mAKAP/CaN. Small molecules might also be potential candidates, since it was already shown that they efficiently disrupt the PPI between CaN and NFAT in T cells (Roehrl et al., 2004).

It has also been shown that mAKAP binds directly to phospholipase C∈ (PLC∈; Zhang et al., 2011), which generates diacylglycerol (DAG) at the nuclear envelope using as substrate phosphatidylinositol 4-phosphate (PI4P) from the Golgi apparatus, leading to activation of protein kinase D (PKD; Zhang et al., 2013). The formation of this complex and its involvement in myocyte hypertrophy has been shown using siRNA based depletion of PLC𝜀 and observing that this prevents development of cardiac hypertrophy (Zhang et al., 2011, 2013). Recently, it was shown that histone deacetylase (HDAC) 4 and PKD, which phosphorylates HDAC4 also resides in this AKAP complex (Kritzer et al., 2014). *In vivo* evidence showed that mAKAP knockout mice have better chance of survival when cardiac hypertrophy is induced both by pressure and catecholamine overload. It was also shown that mAKAP knockout decreased apoptosis, fibrosis and pathological gene expression via decreasing activation/phosphorylation of PLC∈/PKD1/HDAC4 complex proteins (Kritzer et al., 2014). Taken together these data provides a novel therapeutic target between mAKAP/PLC∈ for chronic hypertrophy.

Another potential PPI target is that of the AKAP targeting domain and its localization partner. mAKAP is localized in the perinuclear membrane, however, it is not a transmembrane protein, but binds to the outer nuclear membrane protein nesprin-1α (Pare et al., 2005b). Dodge-Kafka et al. (2005) showed that the supramolecular mAKAP/PDE4D3/Epac/ERK5 complex modulates cardiomyocytes hypertrophy. Briefly, mAKAP anchored PKA phosphorylates PDE4D3 that hydrolyses local cAMP forming a negative feedback loop. At the same time PDE4D3 that binds to Epac1and ERK5 can induce cardiomyocyte hypertrophy. Additionally, RNA interference of mAKAP or disruptor peptides competing for the mAKAP/perinuclear membrane binding site blocks the cytokine induced cardiomyocyte growth (**Figure 3B**) (Dodge-Kafka et al., 2005).

AKAP-Lbc acts as a scaffold protein for several protein kinases: PKA, protein kinase C (PKC) and PKD that phosphorylate different substrates leading to hypertrophy, additionally AKAP-Lbc can also acts as a guanine nucleotide exchange factor (GEF) for the small GTPase Rho (reviewed in Carnegie and Burmeister, 2011; Diviani et al., 2013; Soni et al., 2014).

Unlike the mAKAP complex, PKA binding to AKAP-Lbc leads to myocyte cardioprotection. Recently it was demonstrated that PDE4 directly binds to heat-shock protein of 20 kDa (Hsp20) in the heart (Sin et al., 2011). HSPs are chaperone proteins that are important for normal cell function; moreover, their role in protecting against ischemia-reperfusion injury, apoptosis and hypertrophy is well known (Fan et al., 2006; Edwards et al., 2011; Fan and Kranias, 2011). Edwards et al. (2012) showed the involvement of AKAP-Lbc which anchors PKA and Hsp and is responsible for directing PKA phosphorylation of Hsp20, which is cardioprotective (Lee et al., 2013). In contrast, in the absence of cAMP stimulation PDE4 hydrolyses the basal levels of cAMP and prevents activation of PKA resulting in unphosphorylated Hsp20 (Edwards et al., 2012). Furthermore, targeting the Hsp20-PDE4D interaction with a disruptor peptide reduced the development of pressure overload hypertrophic response in aortic-banded mice (Martin et al., 2014). Moreover, small molecules might offer a potential therapeutic avenue since they have already been shown to modulate Hsp20 activity in human airway smooth muscle (An et al., 2011).

As previously mentioned AKAP-Lbc also recruits PKC and PKD1 which enables phosphorylation and activation of PKD1 by PKC (Carnegie et al., 2004), the activated PKD1 is then released from the complex to phosphorylate HDAC5, which has also been shown to be involved in hypertrophy (Zhang et al., 2002; Vega et al., 2004). Additionally, disruption of AKAP-Lbc/PKD1 interaction by truncating AKAP-Lbc in mouse models affects hypertrophy induced by transverse aortic constriction (TAC)-induced pressure overload (Taglieria et al., 2014).

Finally AKAP-Lbc can act as a GEF for the small GTPase Rho and this signalosome can be a possible target for preventing hypertrophy. It was also shown that due to α-adrenergic receptor stimulation, AKAP-Lbc assembles RhoA effectors PKNα, MLTK, MKK3 leading to activation of p38 MAPK (Cariolato et al., 2011) and that this complex regulates hypertrophic responses in the stressed heart (Pérez López et al., 2013). Furthermore, the authors showed by breeding transgenic mice overexpressing a disruptor peptide that inhibition of AKAP-Lbc/p38 complex reduces cardiomyocyte hypertrophy, proving for the first time an *in vivo* role of AKAP in regulating cardiac hypertrophy (Pérez López et al., 2013).

It is also important to mention that cardiomyocyte hypertrophy may sometimes be beneficial and other times harmful (reviewed in Crozatier and Ventura-Clapier, 2015). In some of the studies mentioned targeting AKAPs decreases compensatory hypertrophy, which can lead to apoptosis and heart failure. Nevertheless, targeting such AKAP complexes may prove useful in combination with other pharmacological approaches.

### (iii) Controlling Electrical Signaling

A-kinase anchoring proteins also play a role in promoting electrical cell-to-cell coupling. Gap junctions play a crucial role in cell-to-cell conductance in cardiomyocytes. These channels are composed of connexin 43 (Cx43) hexamers creating pores throught the cell membrane of two adjacent cells allowing passage of the signal. It has recently been shown that an AKAP, ezrin, is involved in the expression and regulation of gap junction conductivity by organizing a PKA/Cx43/ezrin supramolecular complex in other cell types (Pidoux et al., 2014). Moreover, it is known that Cx43 is involved in several pathological conditions in the heart (reviewed in Severs et al., 2008). For example during ischemia, gap junctions are affected mainly by changes in phosphorylation of Cx43 (Beardslee et al., 2000; Axelsen et al., 2006). Targeting the PPIs between the AKAP and Cx43 in the heart may create a possible therapeutic strategy since Cx43 gap junction communication is damaging in the post-infarction heart (**Figure 3C**).

## Conclusion

Evidence that cAMP compartmentation is particularly important for transmission of accurate and specific biological information is increasing. In order to respond to local cAMP gradients AKAPs contribute to correct and specific propagation of the cAMP signal by organizing supramolecular complexes where PKA, its substrates and other signaling proteins are assembled. These complexes come together through PPIs, which are fundamental for correct propagation of the response. Until recently targeting AKAP interactions by designing peptides to disrupt such binding interfaces was predominantly used as a tool to study function of components of these signalosomes. Currently, there is growing interest in the possibility to develop small molecule PPI disruptors which creates new opportunities for developing therapeutic strategies by preventing interactions in AKAP-complexes. Here we suggest some possible target complexes in the heart, however, other potential targets were not mentioned and more may still be discovered. Finally, we expect that targeting PPIs in complexes organized by AKAPs will receive increased attention as knowledge in development of small molecule PPI disruptors increases and the benefits of specifically perturbing individual signaling complexes come out.

## Conflict of Interest Statement

The authors do research on small molecule PPI disruptors for perturbing AKAP complexes and have patent applications in this area that could constitute future commercial interest. There are no financial relationships that could be construed as a potential conflict of interest.
